# Large-scale population structure and genetic architecture of agronomic traits of garlic

**DOI:** 10.1093/hr/uhad034

**Published:** 2023-02-22

**Authors:** Huixia Jia, Qing Zhao, Jiangping Song, Xiaohui Zhang, Wenlong Yang, Zhenzhen Du, Yue Zhu, Haiping Wang

**Affiliations:** State Key Laboratory of Vegetable Biobreeding, Institute of Vegetables and Flowers, Chinese Academy of Agricultural Sciences, Beijing 100081, China; State Key Laboratory of Vegetable Biobreeding, Institute of Vegetables and Flowers, Chinese Academy of Agricultural Sciences, Beijing 100081, China; State Key Laboratory of Vegetable Biobreeding, Institute of Vegetables and Flowers, Chinese Academy of Agricultural Sciences, Beijing 100081, China; State Key Laboratory of Vegetable Biobreeding, Institute of Vegetables and Flowers, Chinese Academy of Agricultural Sciences, Beijing 100081, China; State Key Laboratory of Vegetable Biobreeding, Institute of Vegetables and Flowers, Chinese Academy of Agricultural Sciences, Beijing 100081, China; State Key Laboratory of Vegetable Biobreeding, Institute of Vegetables and Flowers, Chinese Academy of Agricultural Sciences, Beijing 100081, China; State Key Laboratory of Vegetable Biobreeding, Institute of Vegetables and Flowers, Chinese Academy of Agricultural Sciences, Beijing 100081, China; State Key Laboratory of Vegetable Biobreeding, Institute of Vegetables and Flowers, Chinese Academy of Agricultural Sciences, Beijing 100081, China

## Abstract

Garlic, an asexually propagated crop, is the second important bulb crop after the onion and is used as a vegetable and medicinal plant. Abundant and diverse garlic resources have been formed over thousands of years of cultivation. However, genome variation, population structure and genetic architecture of garlic agronomic traits were still not well elucidated. Here, 1 100 258 single nucleotide polymorphisms (SNPs) were identified using genotyping-by-sequencing in 606 garlic accessions collected from 43 countries. Population structure, principal component and phylogenetic analysis showed that these accessions were divided into five subpopulations. Twenty agronomic traits, including above-ground growth traits, bulb-related and bolt-related traits in two consecutive years were implemented in a genome-wide association study. In total, 542 SNPs were associated with these agronomic traits, among which 188 SNPs were repeatedly associated with more than two traits. One SNP (chr6: 1896135972) was repeatedly associated with ten traits. These associated SNPs were located within or near 858 genes, 56 of which were transcription factors. Interestingly, one non-synonymous SNP (Chr4: 166524085) in *ribosomal protein S5* was repeatedly associated with above-ground growth and bulb-related traits. Additionally, gene ontology enrichment analysis of candidate genes for genomic selection regions between complete-bolting and non-bolting accessions showed that these genes were significantly enriched in ‘vegetative to reproductive phase transition of meristem’, ‘shoot system development’, ‘reproductive process’, etc. These results provide valuable information for the reliable and efficient selection of candidate genes to achieve garlic genetic improvement and superior varieties.

## Introduction

Garlic, an important species in the *Allium* genus (Alliaceae), is consumed as a vegetable and spice for culinary purposes or as an ingredient for traditional and modern medicine [1]. Garlic contains abundant bioactive components like allicin, polyphenols, and sulfur-containing compounds. Through epidemiological studies, garlic has proved to play a role in the prevention of cardiovascular disease, as well as an antioxidant, anti-oxidation, anti-inflammatory, anti-cancer, and anti-pathogen [1, 2]. Thus, garlic has remarkable economic and medicinal values, for which it is cultivated worldwide, with current production of 28 million tons in 1.63 million hectares (FAOSTAT data in 2020). Most of the world’s garlic is produced in Asia. China is the largest producer and accounts for more than 70% of total garlic production.

The cultivation history of garlic dates to approximately 3000 bc in ancient Egypt. Central Asia has been recognized as the primary center of origin, and Mediterranean and Caucasus zones have been considered secondary centers [3]. Although garlic is an asexually propagated crop, it exhibits remarkably extensive diversity in its morphological and botanical traits (e.g. leaf number, bulb weight, clove number, bolting/non-bolting habits, fertility) and phyto-medical component (e.g. allicin content) [4–7]. It has been speculated that this diversity might be the consequence of long-term differentiation and variations from ancestral populations or accumulated mutations throughout garlic cultivation history [8, 9]. The genetic variation of garlic has been evaluated using isozyme loci and molecular markers [10, 11], but to date, the population structure and genetic diversity of large-scale garlic accessions have not been investigated at whole-genome variation level.

Currently, garlic breeding approaches mainly rely on clonal breeding or physical and chemical mutagenesis, which are extremely slow and untargeted [12]. It is difficult to create new varieties through conventional cross- and self-pollination strategies, which seriously hinder breeding process processes. Molecular breeding techniques, including marker-assisted breeding and genetic engineering, are effective tools for accelerating breeding processes. Particularly, gene editing, as an advanced biotechnological technique, enables the precise and efficient targeted modification of the genome, and it has been applied to improve agricultural traits in some important crops [13, 14]. For example, genome editing is a powerful technology in asexually propagated potato to improve important traits such as tuber starch quality, abiotic-biotic resistance, and self-incompatibility [15–18]. These advanced breeding methods may provide opportunities to accelerate trait improvement and varieties innovation of garlic. However, collecting large-scale genotyping data and discovering genome-wide molecular markers further elucidate which loci/genes associated with garlic target traits are prerequisites for molecular breeding [19].

Genome-wide association studies (GWAS), combining high-throughput genome variations and phenotype data, have become a powerful approach to dissect the genetic architecture underlying the phenotypic variation of many plants and identify the potential causative loci/genes [20, 21]. In 2020, genome sequencing and chromosome-level genome assembly of garlic have been accomplished [22], offering the opportunity to perform the sequence alignment of different garlic accessions. Due to the large genome size of garlic (16.2 Gb), genotyping-by-sequencing (GBS) is an effective strategy to identify single nucleotide polymorphisms (SNPs) and conduct population structure analysis and genotype–phenotype association [23,24], with the advantage of high-throughput, speed, and low-cost [25]. Recently, GBS-GWAS has been used to decipher the genetic control of soybean agronomic traits [26], alfalfa yield and nutritional traits [27], pepper capsaicinoid content [28], and grape fruit traits [29]. Furthermore, the lack of sexual reproduction in garlic has restricted the construction of artificial populations and traditional quantitative trait locus mapping. Thus, GBS-GWAS arises as a feasible and efficient method for implementing garlic genotyping and association analysis of its agronomic traits.

Here, we present genotyping results of a collection of 606 garlic accessions worldwide using GBS sequencing. Based on the detected SNPs, we analysed garlic’s population structure and genetic diversity, and conducted GWAS analysis for 20 agronomic traits. The objective of this study was to dissect the genetic architecture of garlic in relation to specific loci/genes associated with agronomic traits.

## Results

### Garlic shows considerable nucleotide variation

To explore large-scale genomic variations, 606 garlic accessions collected from 43 countries, including 450 from Asia, 94 from Europe, 54 from America, and 8 from in Africa, were selected to conduct GBS sequencing (Tables S1 and S2, see online supplementary material). These 606 garlic accessions included 584 *Allium sativum* and 22 *Allium longicuspis* accessions (Table S2, see online supplementary material). A total of 13.4 billion clean reads were obtained, and the average coverage was 3.74%. The sequencing depth to the covering genome was from 2.59 to 15.35×, with an average value of 4.63× (Table S3, see online supplementary material). After quality filtering, 1 100 258 high-quality SNPs were detected, these were homogenously distributed across the garlic genome. The average density was 6.77 SNPs per 100 kb. This low SNP density in genome could be explained by the relative low coverage and sequencing depth. The accuracy of these identified SNPs was estimated to be 98.53% when comparing six pairs of accessions sequenced with low depth (4.70×) and high depth (12.16×; Table S4, see online supplementary material). In these 1 100 258 SNPs, 735 418 were transitions (Ts), and 364 840 were transversions (Tv), with the Ts/Tv ratio of 2.02.

The number of SNPs in eight chromosomes (Chr1–Chr8) ranged from 112 639 to 190 197 SNPs, with an average of 137 532 SNPs per chromosome (Table S5, see online supplementary material). The mean number of homozygous SNPs was 2.55-fold higher than that of heterozygous SNPs. Most of these SNPs (97.36%) were located in intergenic regions, 2.02% were in genic regions, and the remaining 0.62% were in upstream and downstream regions (Table S6, see online supplementary material). Considering those located in the genic regions, 3665 SNPs were in exonic regions, 18 576 SNPs in intronic regions, and 22 SNPs in splicing regions. There were 2116 SNPs (accounting for 57.74% of the SNPs in exonic regions) that generated amino acid mutations, including non-synonymous substitution, stop gain and stop loss (Table S6, see online supplementary material).

### Population genetic structure

**Figure 1:**
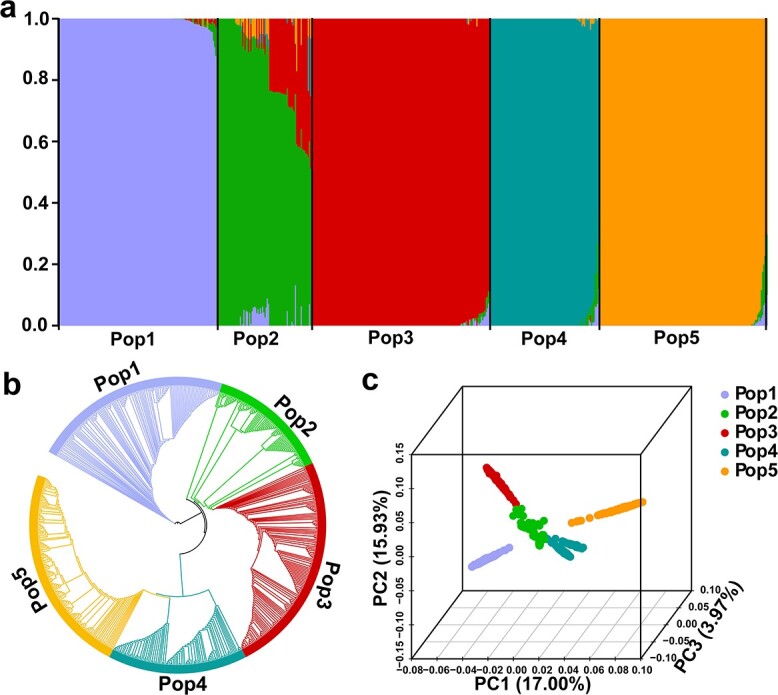
Population structure and phylogenetic analysis of 606 garlic accessions. **a** Population structure of the garlic accessions for *K* = 5. **b** Neighbor-joining tree of the garlic accessions based on the genetic distance. **c** Principal component analysis (PCA) plots showing the first three principal components.

Population structure analysis indicated that the 606 garlic accessions could be divided into five distinct subpopulations (Pop1–Pop5) (Fig. 1a; Table S2, see online supplementary material). Pop1 was composed of 135 *A. sativum* accessions, the vast majority of which (130) were from China (Asia). Four from the remaining five accessions were from Egypt and Uganda in Africa, and one from Greece in Europe.

Pop2 contained 59 *A. sativum* accessions and 22 *A. longicuspis* accessions. *A. sativum* accessions were distributed as follows: 18 from Asian countries including Kazakhstan, Uzbekistan, Georgia, China and Turkey; 21 from Europe, mainly Poland, Germany, Russia, and the Czech Republic; and 20 from the USA. All *A. longicuspis* accessions were from Asian countries comprising Kazakhstan, Tajikistan, Uzbekistan, and Turkmenistan.

Pop3 included 153 *A. sativum* accessions, with the majority (147) from China, Korea, and Myanmar (Asia). Five from the remaining six accessions were from Serbia, Russia, Spain, France, and Portugal (Europe), and one from the USA.

Pop4 was composed of 94 *A. sativum* accessions, with more than a half of the accessions (49) from European countries such as Bulgaria, Poland, Russia, and Spain; 26 from North and South American countries including the USA, Brazil, and Chile; 18 from Asian countries such as China, Pakistan, and Turkey; and the last one from Morocco (Africa).

Pop5 included 143 *A. sativum* accessions distributed across five continents. Most accessions (121) were from Asia (China, Thailand, Syria, India, and Vietnam); 12 were from Europe (Russia, French, and Spain); seven were from North and South America (USA, Honduras, and Brazil); and three from Africa.

The phylogenetic tree showed that Pop2 and Pop3 may be grouped together (Fig. 1b), in line with that Pop2 had a certain amount of genetic component of Pop3 (Fig. 1a). Principal component analysis (PCA) further corroborated the result of the population structure and phylogenetic analysis. The first three principal components explained 36.90% of total variation. Five subpopulations were clearly distinguished, and Pop2 was located in the center of the other four populations (Fig. 1c).

### Population genetic diversity and differentiation

**Figure 2:**
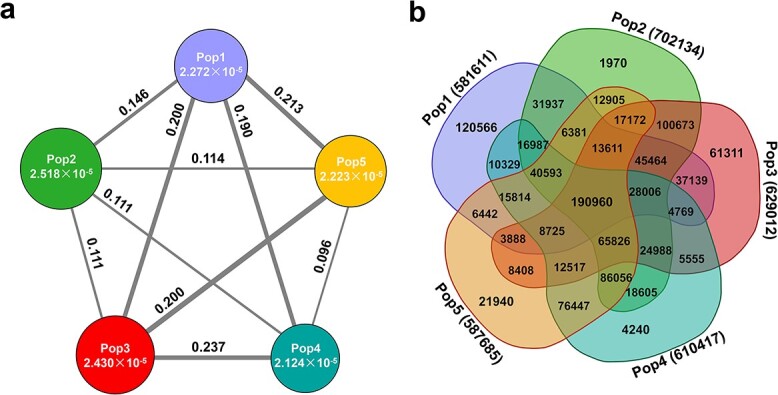
Genetic diversity and differentiation analysis of the five garlic subpopulations*.***a** Nucleotide diversity (*π*) and fixation index (*F_ST_*) across subpopulations. The value in each circle represents the nucleotide diversity for each subpopulation, and the circle size indicates the *π* magnitude; the value on each line indicates the genetic divergence between two subpopulations, and the line width indicates the *F_ST_* magnitude. **b** Venn diagram summarizing the quantity of unique and common SNPs.

The nucleotide diversity (*π*) and fixation index (*Fst*) were calculated to evaluate the genetic diversity of the five subpopulations and their genetic differentiation. Pop2 had the highest nucleotide diversity (*π* = 2.518 × 10^−5^), followed by Pop3 (*π* = 2.430 × 10^−5^), and Pop4 had lowest nucleotide diversity (*π* = 2.124 × 10^−5^) (Fig. 2a). Overall, the genetic diversity of garlic was 2.675 × 10^−5^. The genetic differentiation analysis indicated high differentiation between Pop1 and Pop5 (*F_ST_* = 0.213), Pop1 and Pop3 (*F_ST_* = 0.200), and Pop3 and Pop5 (*F_ST_* = 0.200), and relatively low genetic differentiation between Pop4 and Pop5 (*F_ST_* = 0.096). The genetic differentiation among the other subpopulations ranged from 0.111 to 0.190 (Fig. 2a).

From a total of 1 100 258 SNPs, 190 960 were common SNPs (17.36%) in all five subpopulations, while 210 027 were unique SNPs (19.09%), distributed as follows: 120566 SNPs in Pop1, 1970 SNPs in Pop2, 61 311 SNPs in Pop3, 4240 SNPs in Pop4, and 21 940 SNPs in Pop5 (Fig. 2b). The great number of unique SNPs in Pop1 might explain why this subpopulation clustered into a single branch in the phylogenetic tree. In contrast, Pop2 showed relatively few unique SNPs but many shared SNPs with other subpopulations, explaining why Pop2 was the subpopulation containing the highest level of mixed genetic components (Fig. 2b).

### Phenotypic variation and correlation analysis for agronomic traits

**Figure 3:**
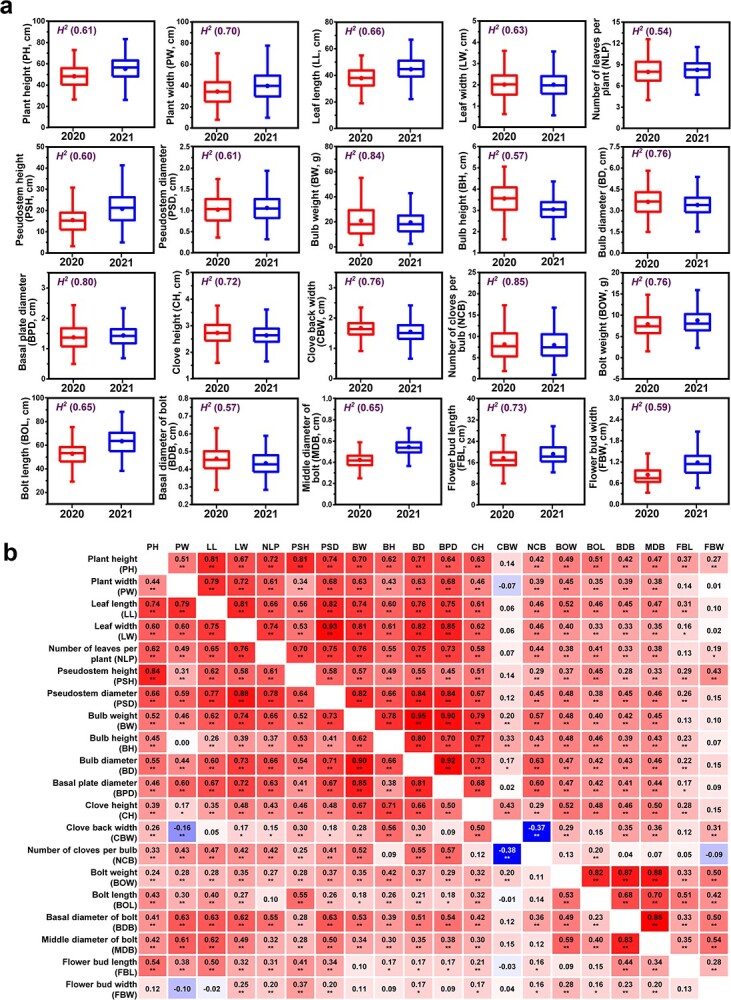
Statistical analysis of 20 garlic agronomic traits. **a** Phenotypic distribution in two consecutive years (2020 and 2021) and broad-sense heritability (*H^2^*) analysis. **b** Heat map of pairwise correlations (Pearson’s *r*) between 2020 (lower triangle) and 2021 (upper triangle) data. Asterisks indicate significant correlations using a two-tailed *t*-test (^*^P < 0.05; ^**^P < 0.01).

For two consecutive years (2020 and 2021), 20 agronomic traits in plants of these 606 garlic accessions were evaluated; they comprised seven above-ground growth traits (plant height, PH; plant width, PW; leaf length LL; leaf width, LW; number of leaves per plant, NLP; pseudostem height, PSH; pseudostem diameter, PSD), seven bulb-related traits (bulb weight, BW; bulb height, BH; bulb diameter, BD; basal plate diameter, BPD; clove height, CH; clove back width, CBW; number of cloves per bulb, NCB), four bolt-related traits (bolt weight, BOW; bolt length, BOL; basal diameter of bolt, BDB; middle diameter of bolt, MDB), and two flower bud traits (flower bud length, FBL; flower bud width, FBW). The detailed descriptive statistics of these traits were showed in Table S7 (see online supplementary material). Extensive variations regarding these traits were found. The BW was the agronomic trait with highest variation, ranging from 1.60 to 60.00 g (coefficient of variation, CV: 58.68%) in 2020 and from 2.50 to 69.33 g (CV: 51.47%) in 2021, followed by the NCB and BOW, suggesting the great potential of these traits for selecting garlic varieties with high-yield or different number of cloves per bulb. In contrast, the CH showed the lowest phenotypic variation, with CVs of 14.98% (2020) and 14.54% (2021). The broad-sense heritability (*H^2^*) for the bulb-related traits, i.e. NCB, BW, and BPD, was high, of more than 0.80, suggesting that these traits were stably inherited and a few environments might be sufficient to assess these traits during screening studies in the search for superior varieties. Oppositely, the *H^2^* of NLP, BH, and BDB was relatively low (0.54, 0.57, and 0.57, respectively), indicating that a larger number of environments would be indispensable for a reliable selection for these traits (Fig. 3a; Table S7, see online supplementary material).

Significant positive correlations were found for the 20 agronomic traits between 2020 and 2021 (Table S7, see online supplementary material). The NCB was the trait with the highest Pearson’s correlation coefficient (*r* = 0.80) on comparing the two years, followed by the BW (*r* = 0.77) and the BPD (*r* = 0.72), further supporting the analysis of broad-sense heritability. The seven above-ground growth traits (PH, PW, LL, LW, NLP, PSH, and PSD) had significant positive correlations with each other (Fig. 3b). The pseudostem was formed from overlapped sheaths of leaves, so the correlation coefficients between LW and PSD were high (*r* = 0.88 and 0.93 in 2020 and 2021, respectively). The BW showed significant positive correlations with the BH, BD, BPD, and CH, whereas the CBW and NCB were significantly negative correlated. Furthermore, most bulb-related traits (BW, BH, BD, BPD, CH, and NCB) demonstrated significant positive correlations with above-ground growth traits, suggesting that above-ground growth traits could be used as predictors of bulb yield of garlic (Fig. 3b).

### Genome-wide association studies for 20 agronomic traits

**Figure 4:**
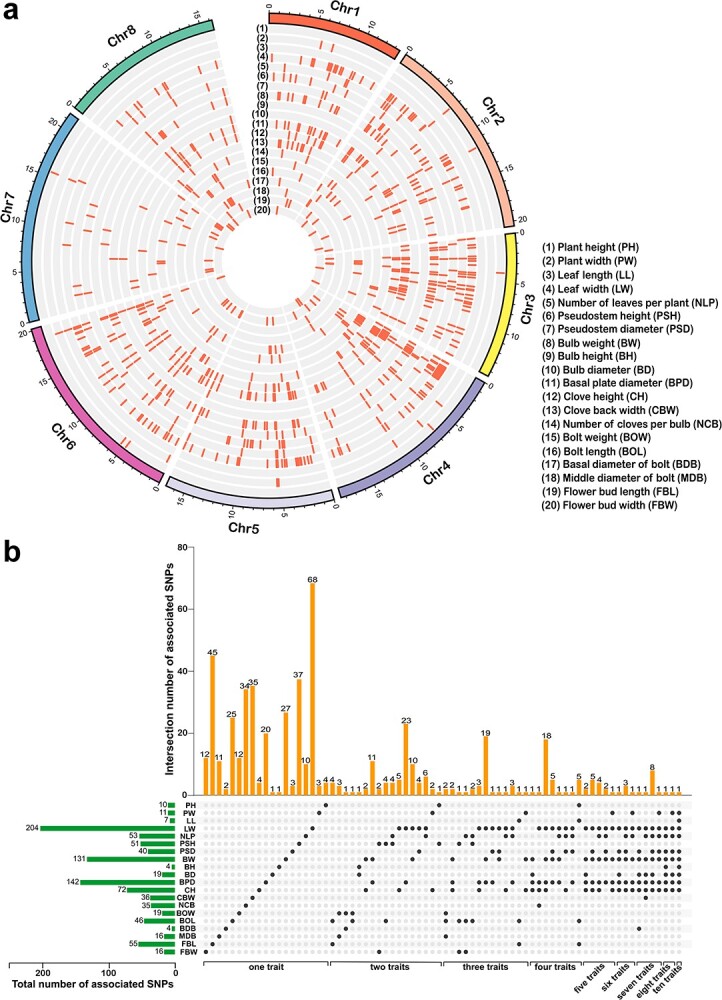
Genome-wide association study of 20 agronomic traits. **a** Circos plot of garlic genome showing SNPs associated with the 20 agronomic traits. The Manhattan plots of each agronomic trait are shown in Figs S1, Figure S3, and S6 (see online supplementary material). **b** Upset Venn diagram showing the overlap number of the SNPs associated with the 20 agronomic traits. The horizontal histogram at the left shows the total number of SNPs in each agronomic trait. The vertical histogram at the top shows the number of unique and common SNPs. The black dots represent the presence of the SNPs associated with the trait that listed in the left side, while the gray dots represent the absence. The vertical column with only one black dot represents unique SNPs in one trait. The vertical column with more than one black dot represents the common SNPs for multiple traits (from two to ten).

To gain insight into the genetic basis of the 20 agronomic traits, the best linear unbiased predictor (BLUP) of each agronomic trait in two consecutive years was subjected to GWAS analysis using mixed linear model (MLM) with correction for population structure effects. With a -log_10_*P* threshold of 6.01, the number of SNPs associated with these 20 agronomic traits varied from four to 204. The BH and BDB had the lower number of associated SNPs, while the LW had the higher number of associated SNPs (Fig. 4; Tables S8 and S9, see online supplementary material). In total, we identified 542 trait-associated SNPs, of which 354 SNPs were associated with only one trait, and 188 SNPs were repeatedly associated with two or more traits (Fig. 4b; Tables S8 and S9, see online supplementary material). Notably, one SNP (chr6: 1896135972) was repeatedly observed in association with ten traits. Among these 542 trait-associated SNPs, 25 were located in genic regions (six non-synonymous SNPs and three synonymous SNPs in exonic regions, 16 SNPs in intronic regions) and 517 in non-coding regions (four SNPs in upstream regions, two SNPs in downstream regions, and 511 SNPs in intergenic regions) (Tables S8 and S9, see online supplementary material). The candidate genes were selected based on the following criteria: (i) those in which the trait-associated SNPs were in genic regions; (ii) those on the two flanks of the trait-associated SNPs in intergenic regions; and (iii) those in which the trait-associated SNPs were in upstream or downstream regions. We found that these 542 trait-associated SNPs were located within or near 858 genes, 56 of which were transcription factors such as *AP2/ERF*, *MYB*, *bHLH*, *TCP*, *WRKY*, *MADS*, *TFIID*, and *TFIIB* (Table S10, see online supplementary material). This GWAS result pointed out important candidate genes to understand regulation mechanisms and achieve the genetic improvement of garlic agronomic traits.

For the seven above-ground growth traits (PH, PW, LL, LW, NLP, PSH, and PSD), 285 SNPs were associated (Fig. S1 and Tables S8 and S9, see online supplementary material). Three SNPs (chr4: 1104921484, chr5: 540826469, and chr6: 1896135972) in intergenic regions were repeatedly associated with PW, LW, NLP, and PSD (Table S9, see online supplementary material). The SNP (chr4: 1104921484) was between *ATP synthase CF0 A subunit* (*Asa4G04013.1*) and *glutathione S-transferase* (*Asa4G04014.1*), the other two SNPs were located near uncharacterized genes (*Asa5G02110.1*, *Asa5G02111.1*, *Asa6G06841.1*, and *Asa6G06842.1*). One non-synonymous SNP (chr4:166524085) repeatedly associated with LW, NLP, and PSD was located in a gene that encoded *ribosomal protein S5* (*RPS5*, *Asa4G00601.1*) (Tables S8 and S9, see online supplementary material). Furthermore, one synonymous SNP (chr4: 1712700119) and two non-synonymous SNPs (chr4: 1712700138 and chr4: 1712700139) associated with LW were located in *Asa4G06220.1* encoding *tetratricopeptide repeats* (*TPR*) superfamily protein.

The bulb is the edible tissue of garlic and an important source of functional components. Diverse bulb phenotypes regarding size, number of cloves, and other morphologic features were observed among these garlic accessions (Fig. S2, see online supplementary material). For seven bulb-related traits (BW, BH, BD, BPD, CH, CBW, and NCB), 268 SNPs were associated (Figure S3 and Tables S8 and S9, see online supplementary material). The non-synonymous SNP (chr4:166524085) in *RPS5* gene was repeatedly associated with the BW, BH, BD, BPD, and CH. Bulb weight is an important factor for garlic bulb yield. There were 131 SNPs associated with BW and located within or near 229 genes (Tables S8–S10, see online supplementary material). These candidate genes included *IAA1-like* (*Asa6G05075.1*), *ERF11-like* (*Asa3G02257.1*), *ERF109-like* (*Asa4G00589.1*), *APETALA1-like* (*Asa6G03209.1*), *mTEF1* (*Asa1G00639.1*), *MYBS1* (*Asa3G03488.1*), and *bHLH18-like* (*Asa6G06851.1*). Based on the published RNA-Seq data [22], gene expression analysis showed that some genes including *snRNP Sm-D2* (*Asa4G00598.1*), *LAP 1-like* (*Asa3G04190.1*), *APETALA1-like* (*Asa6G03209.1*), *probable serine protease EDA2* (*Asa7G00536.1*), *acyl carrier protein 1* (*Asa1G03697.1*), *ERF11-like* (*Asa3G02257.1*), and *DCN1-like* (*Asa5G03370.1*), had high expression levels during bulb development (Fig. S4, see online supplementary material).

**Figure 5:**
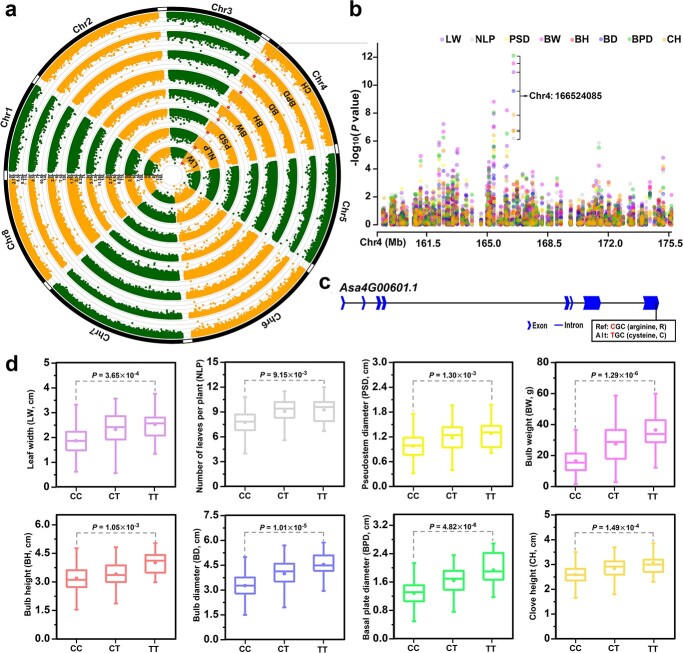
The non-synonymous SNP (chr4:166524085) was associated with both above-ground growth traits and bulb-related traits. **a** Manhattan plot for the GWAS of three above-ground growth traits (LW, NLP, and PSD) and five bulb-related traits (BW, BH, BD, BPD, and CH). The red dot indicates the SNP (chr4:166524085). **b** Partial Manhattan plot of eight traits on chromosome 4 (Chr4). **c** Gene structure of *Asa4G00601.1*. Blue arrows and lines indicate exons and introns, respectively. SNP nucleotide mutation in chr4:166524085 from C to T causes an amino acid substitution from arginine to cysteine. **d** Box plots showing data for these eight traits under three genotypes (CC, CT, and TT). In the box plots, the centerline indicates the median, the box limits are the upper and lower quartiles, and the whisker indicates the 1.5× interquartile range. The statistical significance is analysed with a *t* test.

Notably, the non-synonymous SNP (chr4: 166524085; nucleotide C → T) was repeatedly associated with three above-ground growth traits (LW, NLP, and PSD) and five bulb-related traits (BW, BH, BD, BPD, and CH), with *P* values from 6.65 × 10^−7^ to 7.75 × 10^−13^ (Fig. 5a and b). This SNP was mapped in the first exon of *Asa4G00601.1* that was annotated as *RPS5*, and it gave rise to substitution of amino acid from arginine to cysteine (Fig. 5c). The accessions carrying the TT genotype had significantly higher growth and bulb yield than those with the CC genotype (Fig. 5d), indicating that this variation in the *AsaRPS5* was related to growth and bulb yield. The *RPS5* was highly expressed in bulbs, buds, garlic-sprouts, pesudostems, and leaves (Fig. S5, see online supplementary material), suggesting *AsaRPS5* might be involved in the growth and bulb development of garlic.

**Figure 6:**
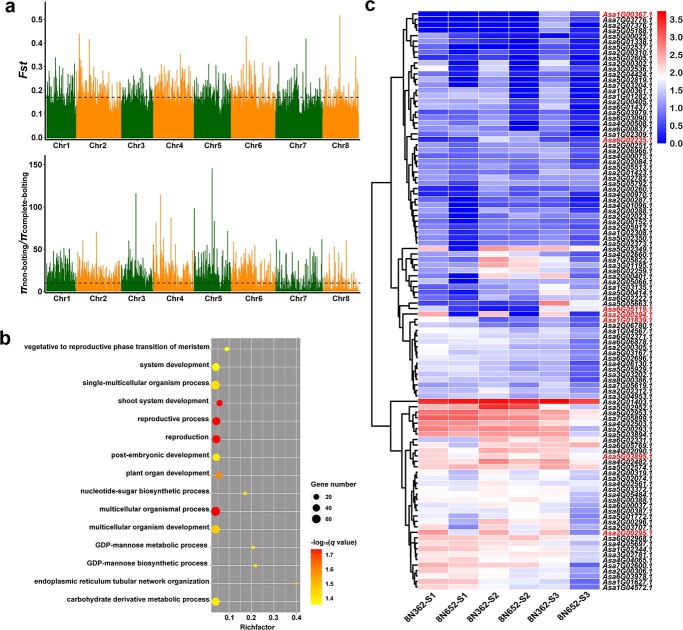
Genomic selection signatures and candidate genes involved in garlic bolting. **a** Genome-wide distribution of *Fst* values and π ratio values between complete-bolting and non-bolting accessions. The black dotted line indicates the thresholds defined by the top 1% of *Fst* values (≥ 0.17) and π_non-bolting_/π_complete-bolting_ values (≥ 9.88). **b** GO annotation of the candidate genes. **c** Heat map of the up-regulated candidate genes in the complete-bolting accession (8 N362) compared with the non-bolting accession (8 N652). The different colors correspond to log_10_ (FPKM+1) values, as shown on the right color scale.

For four bolt-related traits (BOW, BOL, BDB, and MDB) and two flower bud traits (FBL and FBW), 135 SNPs were associated (Fig. S6 and Tables S8 and S9, see online supplementary material). Among these, two SNPs (chr8: 829603022 and chr8: 829603140) were repeatedly associated with the BOW, BOL, and MDB. These two SNPs were between *TFIIS helical bundle-like gene* (*Asa8G03058.1*) and *serine/arginine-rich splicing factor RSZ21A* (*Asa8G03059.1*). Two non-synonymous SNPs (chr7: 2121295307 and chr7: 264535943) were associated with FBL and located in two genes (*Asa7G00898.1* and *Asa7G07646.1*), both annotated as encoding the *activator of 90 kDa heat shock protein ATPase*.

### Genomic selection signatures and candidate genes involved in garlic bolting

Bolting refers to the formation of a flower stalk in garlic plants. Among the 606 garlic accessions used in this study, 328 accessions showed complete-bolting, 103 accessions showed incomplete-bolting, and 175 accessions showed non-bolting (Table S2, see online supplementary material). These three bolting types were distributed across the five continents, with more than half of Asian accessions being the complete-bolting type (Table S2, see online supplementary material).

To identify potential selection signatures and candidate genes involved in bolting, the 328 complete-bolting accessions and 175 non-bolting accessions were scanned genomic regions using *Fst* and *π* (Fig. 6a). The regions with the top 1% cut-off (*Fst* ≥ 0.17 or π_non-bolting_/π_complete-bolting_ ≥ 9.88) were studied as potential regions under selection. In total, 627 candidate genes were identified in the top 1% windows with high *F_ST_* values, and 685 candidate genes were identified in the top 1% windows with high π_non-bolting_/π_complete-bolting_ values, totalizing 1238 candidate genes, of which 74 were identified repeatedly by both methods (Table S11, see online supplementary material). Gene ontology (GO) enrichment analysis showed that these genes were enriched in multiple terms related to reproductive growth, such as ‘vegetative to reproductive phase transition of meristem’, ‘shoot system development’, or ‘reproductive process’ (Fig. 6b).

To compare the differences in the expression profiles of these 1238 candidate genes, one representative complete-bolting accession (8N362) and one representative non-bolting accession (8N652) were subjected to transcriptome sequencing. We detected 186 differentially expressed genes between 8N362 and 8N652, including 106 up-regulated genes (Fig. 6c) and 80 down-regulated genes (Fig. S7, see online supplementary material). For instance, *flowering-promoting factor 1-like protein 1* (*FLP1*, *Asa6G02235.1*) had higher expression levels in 8N362 than 8N652 at the flower bud differentiation stage (S2). *GATA transcription factor 22* (*GATA22*, *Asa6G05119.1*) and *auxin transporter-like gene 2* (*AUX2-like*, *Asa1G01839.1*) had higher expression levels in 8N362 than in 8N652 at before sowing (S1) and bolting stages (S3). *AGL6-like MADS-box gene* (*Asa1G00367.1*) showed higher expression levels in 8N362 than 8N652 at bolting stages (S3). The expression of three histone H3 members (*Asa2G00294.1*, *Asa2G00295.1*, and *Asa5G03895.1*) was significantly higher during three stages in 8N362 than 8N652, whereas the H2A (*Asa4G02276.1*) showed the opposite tendency, with higher expression levels during three stages sampled in 8N652 than in 8N362.

## Discussion

*A. longicuspis* believed as the ancestor of *A. sativum* might not be a separate species. The morphological and karyological characters are similar in *A. sativum* and *A. longicuspis*. Previous researches point out that the filaments of *A. sativum* are typically shorter than the perianths, while *A. longicuspis* has exserted anthers and is found in the center of origin of garlic [3, 30]. Thus, *A. longicuspis* has been considered as an ancestral type. However, exserted anthers have also been observed in *A. sativum* clones with fertile flowers [9], and *A. longicuspis* do not always open flowers [30]. Furthermore, the results from karyotype, isozyme, RAPD, and AFLP analysis demonstrate that *A. longicuspis* variation lies within the range of the variation observed among garlic accessions [4, 31, 32]. Based on these results, some researchers propose that *A. longicuspis* is not a separate species but rather a subgroup or subspecies of *A. sativum* [3, 4, 31]. In line with this hypothesis, our results showed that 22 *A. longicuspis* accessions were clustered in the Pop2, and they had no clear separation from *A. sativum* accessions, suggesting that *A. longicuspis* was not genetically distinct from *A. sativum.*

Although garlic has been cultivated since approximately 5000 years ago, its center of origin has been unclear for a long time. At present, Central Asia is considered as the primary center of origin due to the discovery of primitive fertile plants of garlic in the northwest side of the Tien Shan Mountains in Central Asia [9], and because most variation was found among Central Asian accessions using biochemical and molecular markers [33]. In our study, the Central Asian accessions from Uzbekistan, Kazakhstan, Tajikistan, and Turkmenistan were dispersed in Pop2, Pop4, and Pop5, while the East Asian accessions, mainly from China, were dispersed among five subpopulations, indicating that more abundant genetic components took place in East Asia compared to Central Asia. We speculated that this might result from a distinctly larger number of accessions in East Asia compared with Central Asia in this study. In addition, China has carried out an extensive collection of resources, and new varieties have been cultivated in recent decades, so many genotypes might be found in nature or have been generated through mutation or domestication to adapt to the environment and consumers’ demands. Previous research reported that Mediterranean and Caucasus Zones could be secondary origin centers of garlic [3, 36]. Our result showed that the accessions in Mediterranean zone possessed the genetic components of all five subpopulations, further suggesting that Mediterranean zone could be an important garlic origin center of garlic.

Exploring candidate genes involved in controlling target traits can facilitate genetic improvement of garlic through marker-assisted breeding or genetic manipulation. The reproductive ability of garlic has been lost during selective domestication for large bulbs, and the commercial garlic cultivars grown nowadays are generally sterile and cannot produce seeds [3]. Although two garlic genetic maps have been published [34, 35], the small population size and low density of markers might limit the further quantitative trait loci mapping for traits. Recently, the genetic basis of clove shape traits (clove length, width, and thickness) and bulb yield traits (bulb weight, diameter, and number of cloves) has been assessed through a transcriptome-referenced association study, and dozens of trait-associated transcripts, including long non-coding RNAs and genes, were screened out to provide a reference for improvement of bulb yield in garlic breeding [36, 37]. Moreover, the diversification of bulb traits has been analysed through selective sweeps, genome-wide trait association studies, and associated transcriptomic analysis [12]. However, the genetic architecture of garlic agronomic traits has not been studied in-depth using large-scale accessions, and lacking reliable regulatory genes hinders the parsing of molecular mechanisms. In this study, 858 candidate genes associated with 20 garlic agronomic traits were identified, providing novel insights into their genetic basis.

The non-synonymous SNP (chr4:166524085) in the *RPS5* (*Asa4G00601.1*) gene was repeatedly associated with above-ground growth and bulb-related traits. As a component of the ribosomal 40S subunit, RPS5 protein not only participates in protein synthesis and processing, but also has some extraribosomal functions [38]. In humans, *RPS5* gene participates in a variety of pathological processes, especially in tumorigenesis and cell transformation, and germ cell and follicle cell differentiation [39, 40]. In *Arabidopsis*, the homozygous mutant of *AtRPS5* causes embryo lethality and reduces gamete viability, while the heterozygous mutant shows slow growth and defective flower and vascular tissues [41]. In this study, the *AsaRPS5* gene was associated with growth and bulb-related traits, and it had high expression level in bulbs, buds, garlic-sprouts, pesudostems, and leaves of garlic. Therefore, the *RPS5* gene could be considered an important candidate gene to increase garlic yield through marker screening and genetic engineering in the near future.

Bolting is necessary for garlic plants to achieve flowering and reproductive growth. In this research, we differentiated among complete-bolting, non-bolting, and incomplete-bolting garlic accessions [30]. In the complete-bolting type, the apical meristem of the scape differentiates floral primordial interspersed with bulbil primordia [42]. Through genetic differentiation analysis between complete-bolting and non-bolting accessions, 1238 candidate genes were screened and found to be enriched in multiple GO terms related to reproductive growth, especially ‘vegetative to reproductive phase transition of meristem’. Some candidate genes such as *FLP1* (*Asa6G02235.1*), *GATA22* (*Asa6G05119.1*), *AGL6-like MADS-box gene* (*Asa1G00367.1*), and *histone H3* (*Asa2G00294.1*, *Asa2G00295.1*, and *Asa5G03895.1*) showed higher expression levels in the complete-bolting accession than the non-bolting accession. *FLP1*, a homologous gene of *flowering promoting factor 1* (*FPF1*), has been shown to play roles in floral induction and flowering time control in *Arabidopsis* [43]. Although the function of *GATA22* is poorly known, other members of *GATA* transcription factors have been demonstrated to participate in flowering induction and flower development. For instance, a GATA-3-type transcription factor *HANABA TARANU* (*HAN*) bridges meristem and organ primordia boundaries through *PINHEAD*, *JAGGED*, *BLADE-ON-PETIOLE2*, and *CYTOKININ OXIDASE 3* during flower development [44]. The *han* mutants have reduced numbers of petals and stamens and fused sepals in *Arabidopsis* [45]. *AGL6-like gene*, which encodes typical MIKC-type MADS box protein expressed in floral tissues, has been demonstrate to regulate flowering time, floral organ identity, meristem fate, and ovule (integument) and seed development in wheat, rice, and *Arabidopsis* [46–48]. Histone H3 is one of the core components of the nucleosome. Its N-terminal tail may undergo various post-translational modifications relevant to sexual reproduction regulation. Among them, it may be mentioned the trimethylation of histone H3 at lysine 4 (H3K4), repressing the floral transition [49], or phosphorylation of histone H3 at threonine 3 (H3T3), promoting flowering [50]. Furthermore, the disruption of either EARLY BOLTING IN SHORT DAY (EBS)-H3K27me3 or EBS-H3K4me3 interaction induced early floral transition in Arabidopsis [51]. The high expression levels of three histone H3 members in the complete-bolting accession might imply that more histone H3 products were modified to prompt bolting in garlic. Considering their function of orthologous genes in other species and expression patterns, these genes could be regarded as potential candidate genes to regulate bolting and floral development in garlic.

## Materials and methods

### Garlic materials and GBS sequencing

A total of 606 garlic accessions from 43 countries were selected to conduct GBS sequencing and phenotypic measurements (Tables S1 and S2, see online supplementary material). The young leaves of the 606 accessions (at 4–6 leaf stage) were sampled and stored at −80°C. Genomic DNA was isolated using the cetyltrimethylammonium bromide (CTAB) method. The DNA concentration and quality were detected using agarose gels and Nano-photometer. After passing the quality testing, the extracted DNAs were digested using two restriction enzymes (*EcoR* I and *NIa* III) to construct a pair end library with a length range of 400–600 bp. The library was sequenced on the Illumina Novaseq 6000 platform at Shanghai Biozeron Biotechnology Co., Ltd (Shanghai, China). Finally, a total of 14.8 billion PE150 reads were obtained.

### SNPs calling and functional annotation

The raw reads were subjected to quality control and filtered to obtain clean reads. Quality control procedures were implemented to remove the reads as follows: (i) adapter sequence; (ii) PCR duplicate reads; (iii) read quality score <20; (iv) reads containing N >10%; and (v) read length after discarding adapter and quality trim <75 bp. Subsequently, the clean reads were aligned to the garlic genome [22] using the Burrows-Wheeler Aligner program (BWA, version 0.7.8-r455) [52] with the command ‘mem -t 4 -k 32 -M’. The alignment results were converted to BAM file using SAMtools (version 0.1.19-44 428 cd) [53]. For SNPs calling, we used a Bayesian approach as implemented in the genome analysis toolkit HaplotypeCaller (version 4.0.10.1). After further filtration, the SNPs (minor allele frequency ≥0.05, mapping quality ≥20, and missing rates <0.20) were retained for subsequent analysis. The ANNOVAR package was used for SNP annotation [54].

### Population genetics analysis

The genetic structure and estimated admixture proportions were investigated using the ADMIXTURE software (version 1.3.0) [55]. The number of genetic clades was predefined from *K* = 1 to 10. The most suitable number of *K* was selected according to the lowest cross validation (CV) error. PCA analysis was performed to evaluate genetic structure using the GCTA software (version 1.93.2) [56]. To clarify the phylogenetic relationships, a neighbor-joining tree was constructed based on the *P*-distance using the TreeBeST software (version 1.9.2) and visualized using iTOL (version 6.0). Nucleotide diversity (*π*) was used to estimate the degree of variability within each subpopulation, and fixation index (*F_ST_*) among subpopulations was used to explain genetic differentiation. The *π* and *F_ST_* were calculated using VCFtools (version 0.1.14) [57].

### Field experiments and phenotypic measurements

The 606 garlic accessions were planted in the experimental field at the Vegetable Research Center of International Agricultural High and New Technology Industrial Park, Chinese Academy of Agricultural Sciences. In October 2020 and 2021, 50 cloves per accession with uniform size were sown in a plastic house, separated by 10 cm in rows and 20 cm in columns. The experiments were conducted using a complete randomized block design with three replicates. The field management was carried out according to normal cultivation practices. Five plants per each accession were randomly selected for evaluation of the 20 agronomic traits, including seven above-ground growth traits, seven bulb-related traits, four bolt-related traits, and two flower bud traits. These agronomic traits were measured according to the garlic descriptors developed by the International Plant Genetic Resources Institute [58] and the garlic descriptors and data standard [59]. The PH, PW, LL, LW, PSH, PSD, and BOL were measured with a ruler. The BH, BD, BPD, CH, CBW, BDB, MDB, FBL, and FBW were measured with a vernier caliper. The BW and BOW were weighed using an electronic balance. The NLP and NCB were counted artificially. The bolting habit was divided into three types: complete-bolting, incomplete-bolting, and non-bolting.

### Phenotyping statistical analysis

The two-year phenotypic dataset was analysed using the IBM SPSS software (version 19.0) to generate descriptive statistics. Pearson’s correlation coefficient was calculated to examine correlations between the agronomic traits. The *H^2^* of each trait was estimated using the lme4 package (version 1.1–27.1) of the R program (version 4.0.3) under the following formula: *H^2^* = δ^2^g/(δ^2^g + δ^2^ge/n + δ^2^/nr). In this equation, δ^2^g represents the genetic variance, δ^2^ge represents the interaction variance of genotype × environment, δ^2^ represents the error variance, n represents the number of environments, and r represents the replications per environment. The BLUP of each trait was also calculated by the lme4 package [60].

### Genome-wide association analysis of agronomic traits

The detected SNPs were subjected to GWAS for the 20 agronomic traits. MLM analysis was carried out using GEMMA (version 0.98.1) [61] under the following equation: *y* = *Xα* + *Sβ* + *Kμ* + *e*. In this equation, *y* represents the phenotype, *X* represents the SNP genotype, *S* represents the structure matrix, *K* represents the relative kinship matrix, *Xα* and *Sβ* represent fixed effects, and *Kμ* and *e* represent random effects. The top three principal components were used for population structure correction. The *P* value threshold for significance was estimated using 1/n [62–64]. The n is the effective number of independent SNPs, which was calculated using GEC software [65]. The value for n was 1 028 952, and the *P* value threshold for significance was 9.72 × 10^−7^ [−log_10_ (*P* value) = 6.01].

### Analysis of genomic selection signatures and identification of candidate genes involved in bolting

To identify potential selection signatures and candidate genes involved in bolting, *Fst* and *π* were measured between the complete-bolting accessions and non-bolting accessions with VCFtools (version 0.1.14) [57], setting a sliding window of 120 kb with a step size of 10 kb. Genomic windows with the top 1% windows with high *Fst* and *π* ratio values were selected as potential selection regions. The genes in the potential selection regions were considered as candidate genes involved in bolting. Gene ontology enrichment of these candidate genes was performed using Blast2GO (version 5.2.5).

### Expression level analysis of candidate genes

To estimate the expression profiles of the candidate genes involved in bolting, one representative complete-bolting accession (8N362) and one representative non-bolting accession (8N652) were selected for transcriptome sequencing. In 8N362, stem apex before sowing (S1), flower bud at flower bud differentiation (S2) and flower bud at incipient bolting (S3) were sampled for RNA-Seq. In 8N652, stem apex at the same three time-points were sampled. Three biological replicates were performed for each sample. Total RNA was isolated using RNA extraction kit (Huayueyang, China). The RNA quality and quantity were detected by agarose gels, Nano-photometer, and Agilent 2100 bioanalyzer. A total of 18 RNA-Seq libraries were prepared using NEBNext Ultra Directional RNA Library Prep Kit for Illumina. The libraries were sequenced using Illumina HiSeq 2500 platform. The expression profiles of candidate genes associated with bulb-related traits and above-ground growth traits were analysed using the published RNA-Seq data during bulb development process and across different tissues [22].

After filtering adapter sequences and low-quality reads, the clean reads were aligned to the garlic genome using Bowtie2 (version 2.4.4) [66]. Gene expression levels were calculated using Cufflinks (version 2.2.1) [67]. The differentially expressed genes were identified by DESeq (Version 1.18.0), with threshold for false discovery rate ≤0.05 and absolute value of log_2_ (fold change) ≥1. Heat map was plotted using the pheatmap package (version 1.0.12) in R software.

## Acknowledgments

This work was financially supported by the Natural Science Foundation of China (31872946, 32172566, and 32272731), National Key R&D Program of China (2021YFD1200201), China Agriculture Research System of MOF and MARA (CARS-24-A-01), Science and Technology Innovation Project of Chinese Academy of Agricultural Sciences (CAAS-XTCX2018021), Youth Innovation Special Task of Chinese Academy of Agricultural Sciences (Y2023QC06), Agricultural Basic Long-Term Scientific and Technological Work (NAES-GR-005), Safe Preservation Project of Crop Germplasm Resources of MOF (2022NWB037) ,and National Hoticultural Gerplasm Centre Project (NHGRC2022-NH01).

## Author contributions

H.J. and H.W. conceived and designed the research. J.S., X.Z., W.Y., and H.W. prepared the materials. Q.Z., J.S., X.Z., W.Y., Z.D., and Y.Z. performed field experiments and phenotypic determination. H.J., Q.Z., and H.W. performed sampling, sequencing, and data analysis. J.S., X.Z., W.Y., and H.W. contributed to the project discussion. H.J. wrote the manuscript. H.W. revised the manuscript.

## Data availability

The raw data of genotyping-by-sequencing of 606 garlic accessions have been deposited in the Genome Sequence Archive in the National Genomics Data Center (https://bigd.big.ac.cn) under accession number CRA007913. The raw data of transcriptome sequencing of 8N362 and 8N652 have also been deposited in the GSA with accession number CRA007870.

## Conflict of interest statement

The authors declare that they have no conflicts of interest.

## Supplementary data

Supplementary data is available at *Horticulture Research* online.

## Supplementary Material

Web_Material_uhad034
