# Supplementary figures and images for: Large-scale population structure and genetic architecture of agronomic traits of garlic
Source: Hortic Res. 2023 Feb 22;10(4):uhad034. doi: 10.1093/hr/uhad034 (PMC10548411; doi:10.1093/hr/uhad034)

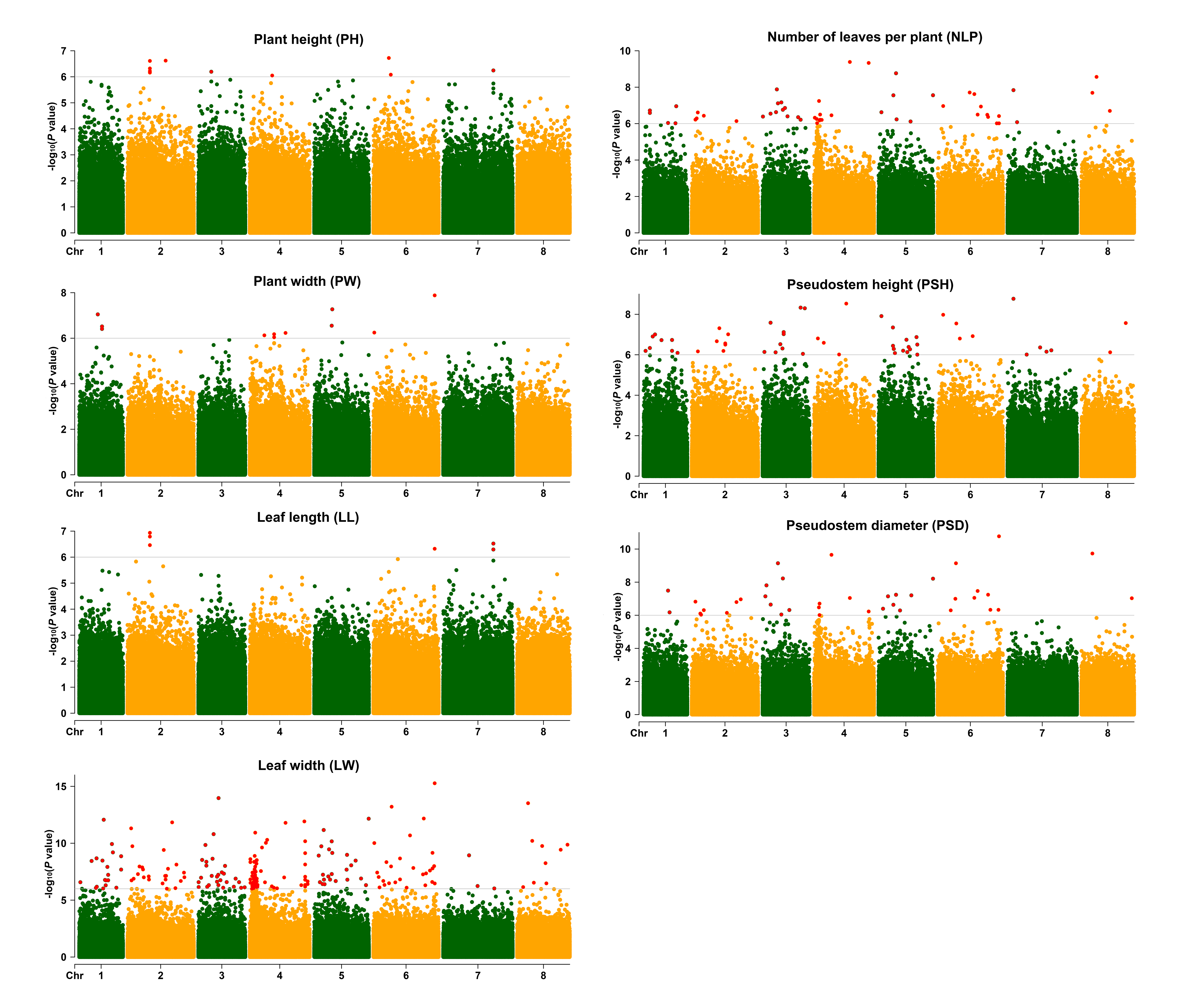

Supplement: Web_Material_uhad034 [file web_material_uhad034.zip › Figure S1.TIFF]

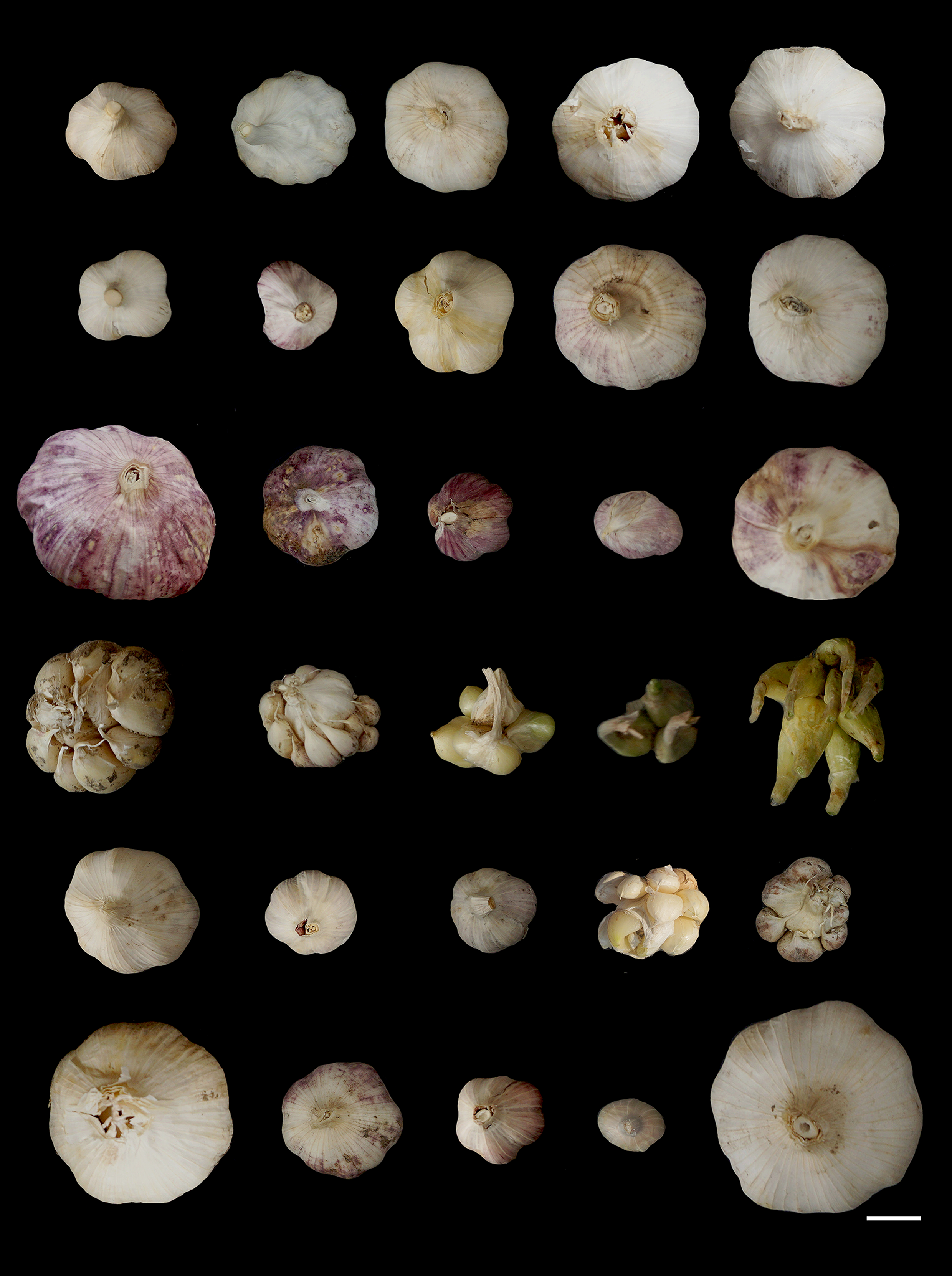

Supplement: Web_Material_uhad034 [file web_material_uhad034.zip › Figure S2.TIFF]

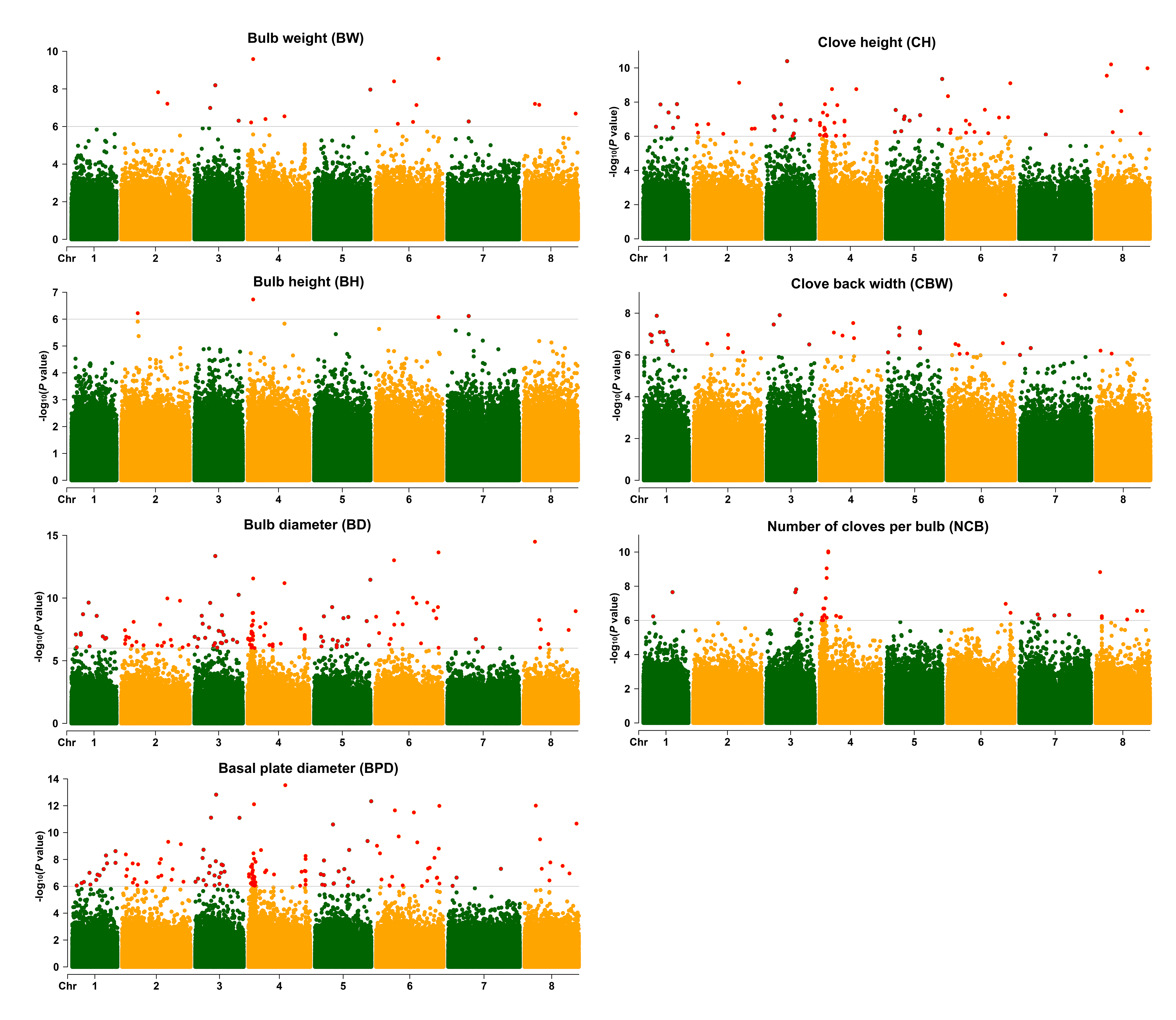

Supplement: Web_Material_uhad034 [file web_material_uhad034.zip › Figure S3.TIFF]

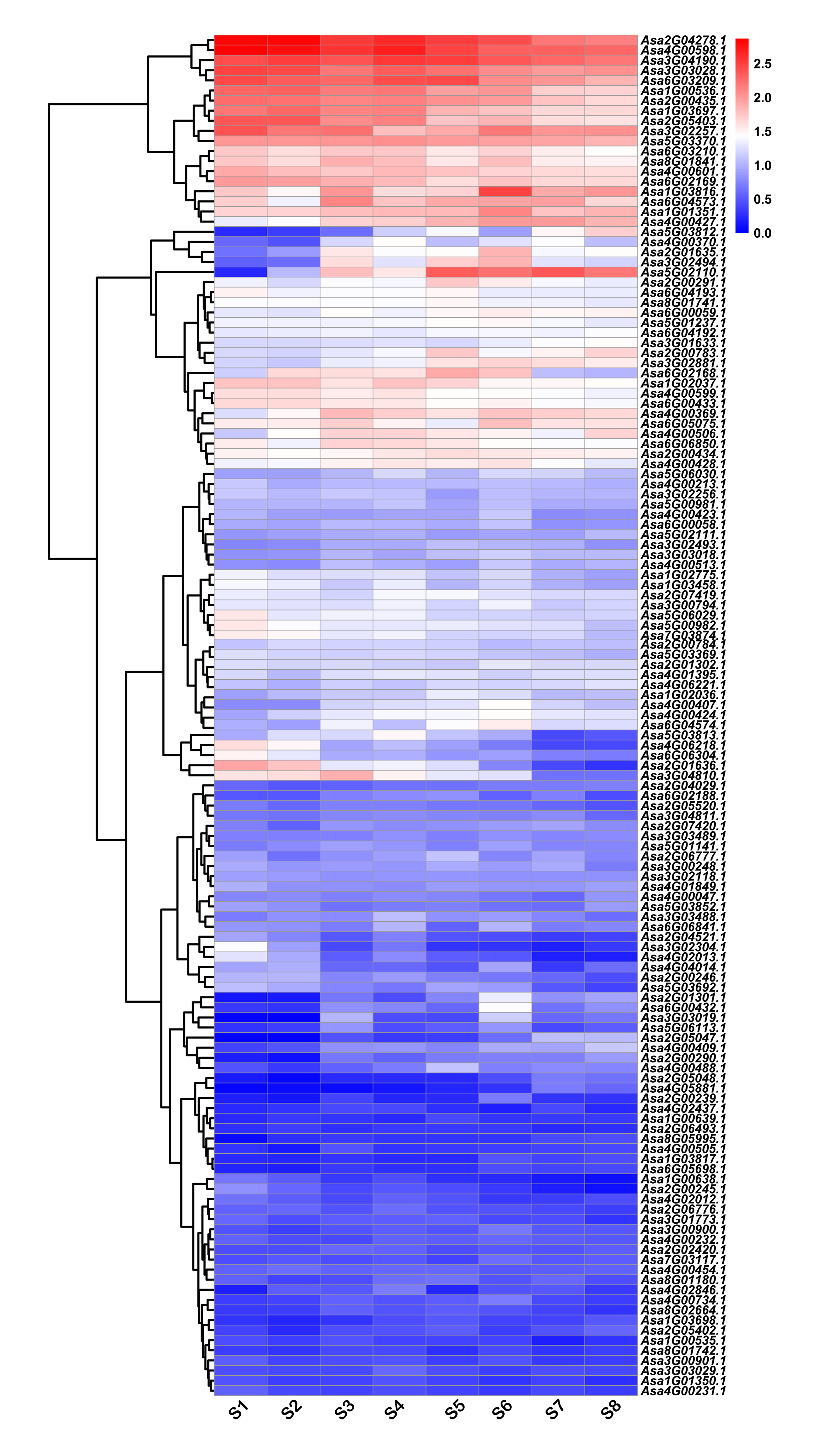

Supplement: Web_Material_uhad034 [file web_material_uhad034.zip › Figure S4.TIFF]

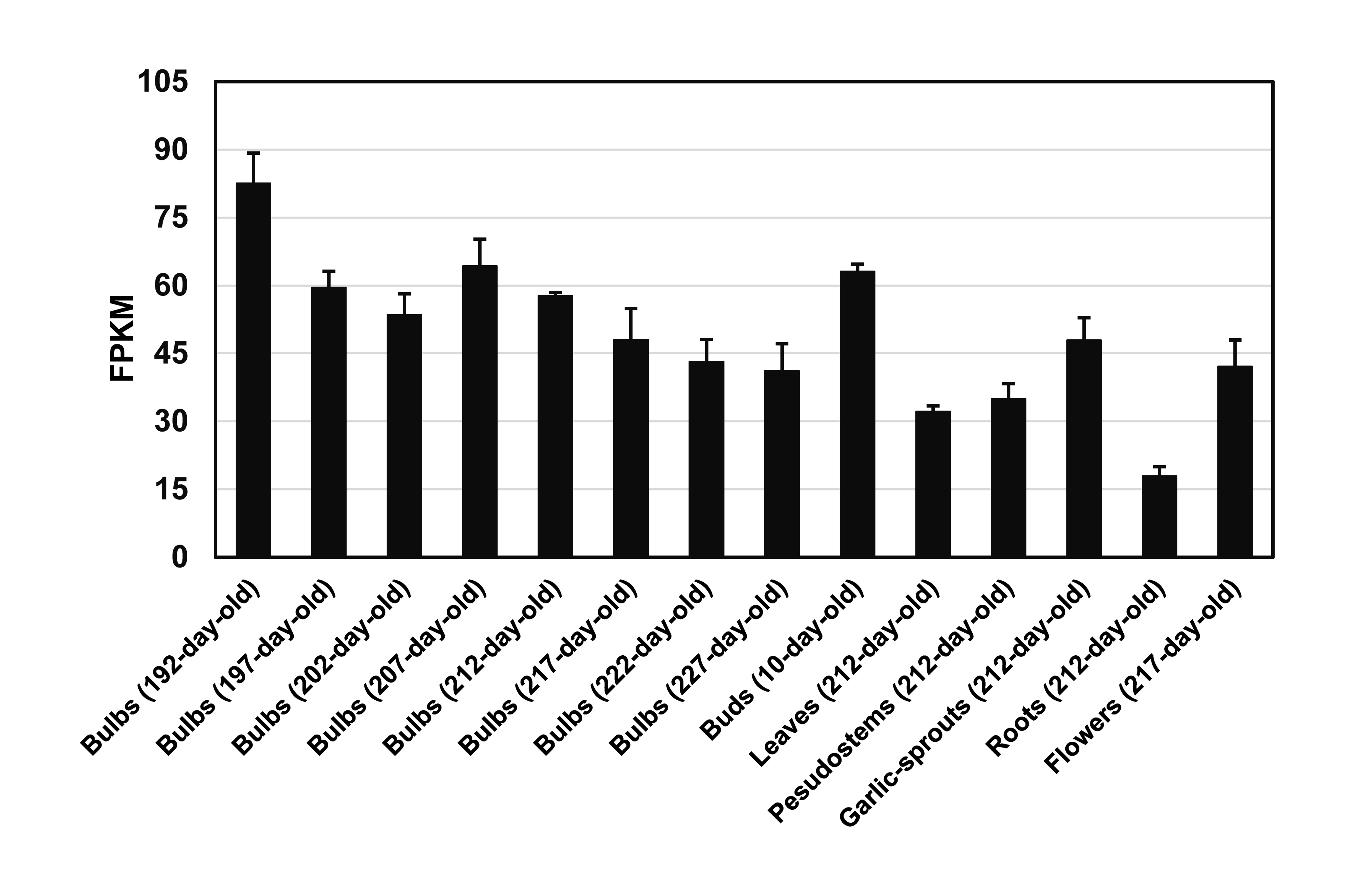

Supplement: Web_Material_uhad034 [file web_material_uhad034.zip › Figure S5.tiff]

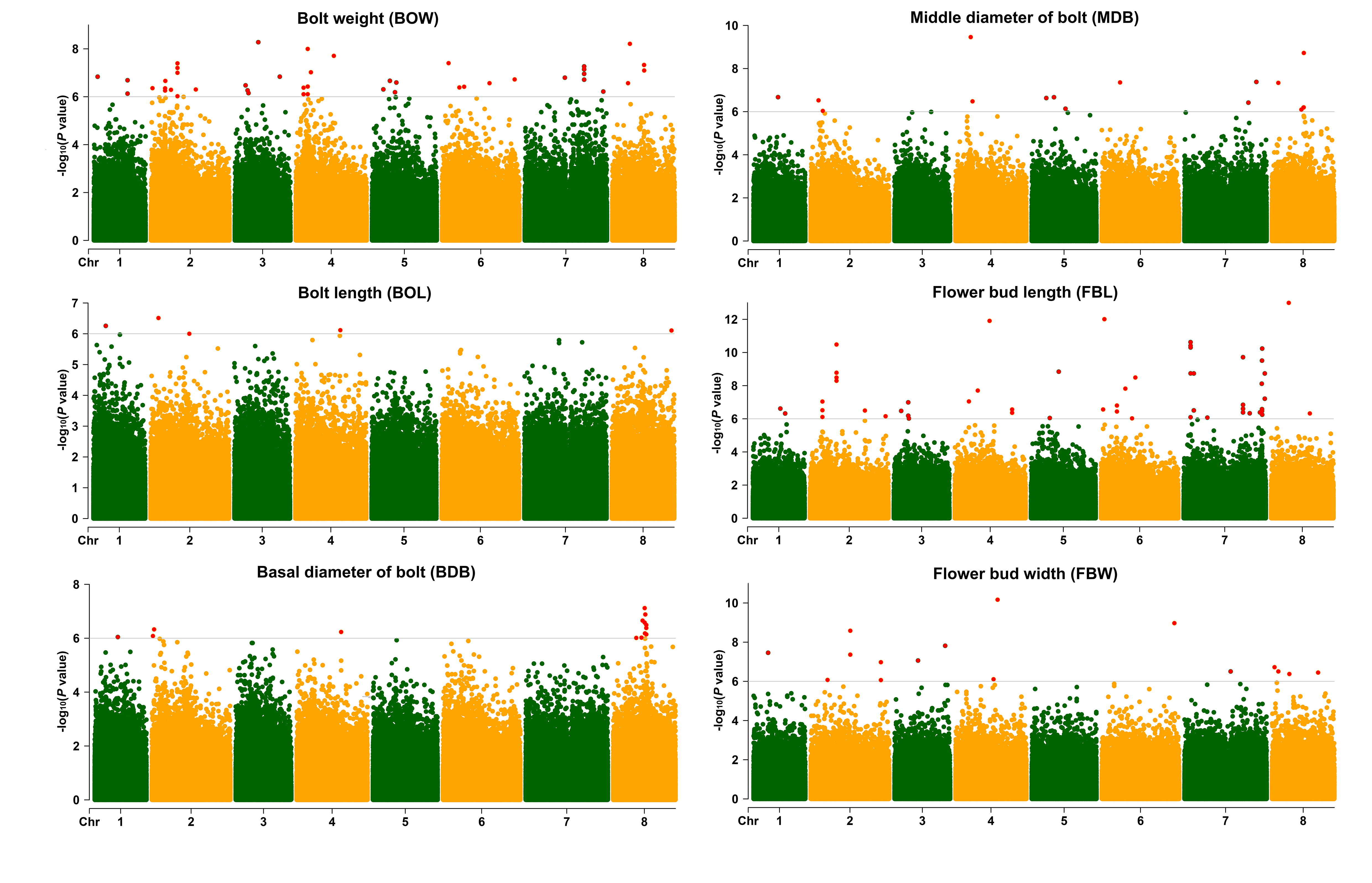

Supplement: Web_Material_uhad034 [file web_material_uhad034.zip › Figure S6.TIFF]

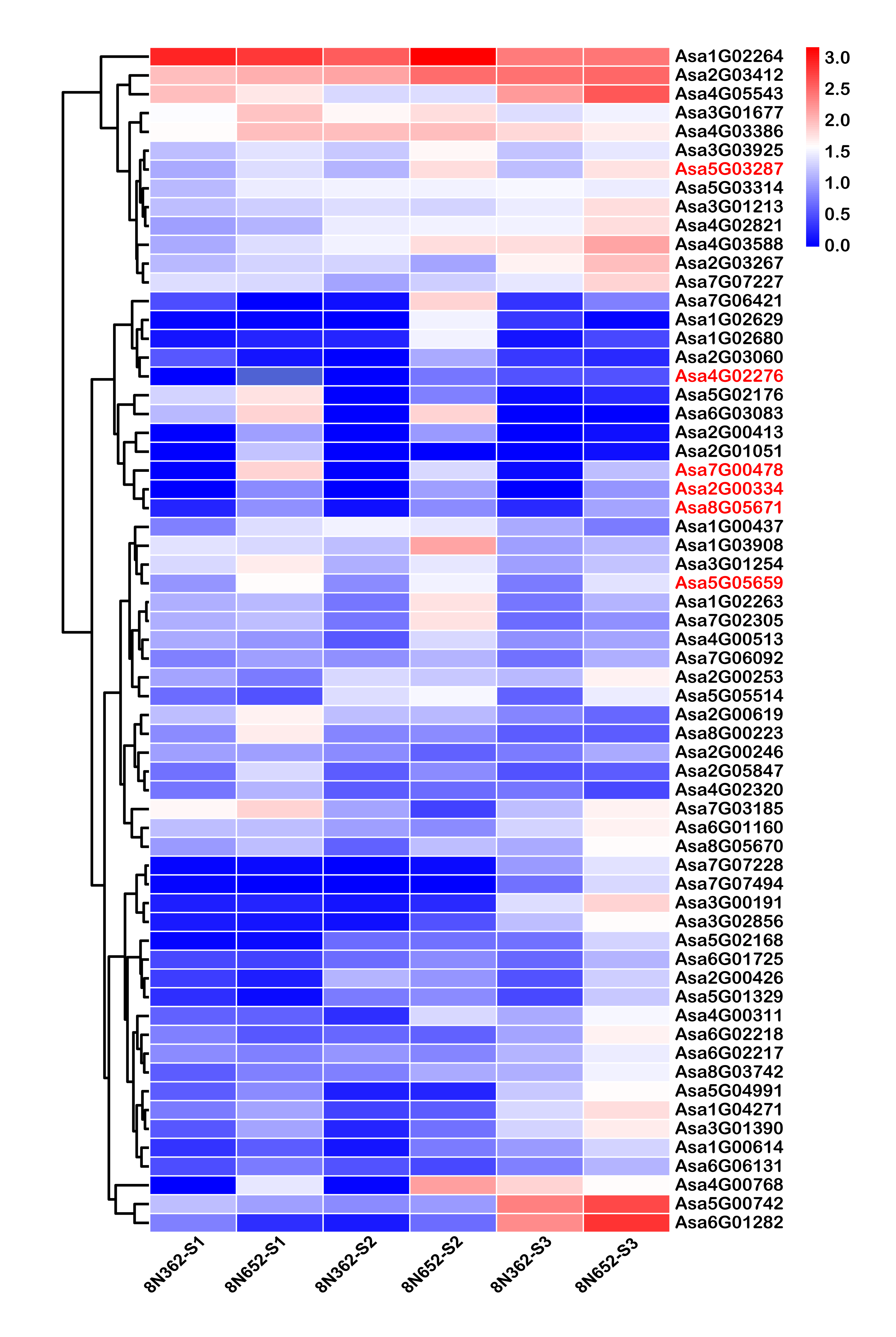

Supplement: Web_Material_uhad034 [file web_material_uhad034.zip › Figure S7.TIFF]
